# Transcriptomics, identification, and testing of manganese metabolism-related genes in cervical squamous cell carcinoma

**DOI:** 10.3389/fonc.2026.1909880

**Published:** 2026-09-15

**Authors:** Wen-Ji Fang, Yi-Fan Zhang, Jie Zhang, Tao Zhang, Xiang Zhang

**Affiliations:** 1 Department of Oncology, Key Laboratory of Immunity, Inflammation & Cancer (Chongqing Municipal Health Commission), The First Affiliated Hospital of Chongqing Medical University, Chongqing, China; 2 Department of Neurology, School of Basic Medical Sciences, Chongqing, China; 3 Department of Neurology, The First Affiliated Hospital of Chongqing Medical University, Chongqing, China; 4 Key Laboratory of Major Brain Disease and Aging Research(Ministry of Education), Chongqing Medical University, Chongqing, China

**Keywords:** bioinformatics, cervical squamous cell carcinoma, drug sensitivity, immune infiltration, key genes, manganese metabolism, prognostic risk model

## Abstract

**Background:**

Cervical squamous cell carcinoma (CSCC) remains a leading cause of cancer-related mortality in women, and few effective prognostic biomarkers have been identified. Although manganese metabolism (MAM) has been implicated in tumorigenesis and immune regulation, its prognostic relevance in CSCC has not been systematically explored, and existing prognostic models for CSCC have largely ignored MAM-related genes.

**Methods:**

CSCC transcriptomic and clinical datasets were obtained from public databases. MAM-related differentially expressed genes (DEGs) were identified by intersecting DEGs, weighted gene coexpression network analysis key module genes, and a curated MAM gene set. Prognostic genes were screened using univariate Cox, least absolute shrinkage and selection operator, and multivariate Cox regression analyses to construct a risk model. The model’s performance was assessed *via* Kaplan–Meier survival analysis, time-dependent receiver operating characteristic curves, and external validation. Associations with immune infiltration, drug sensitivity, and clinical features were further investigated. Experimental validation was performed using real-time quantitative polymerase chain reaction, western blotting, and immunofluorescence staining in CSCC and normal cervical cells.

**Results:**

A four-gene prognostic signature (*PARP1*, *SLC20A1*, *GALNTL6*, and *TAGLN*) was established. Patients were stratified into high- and low-risk groups, and survival was significantly worse in the high-risk group (p = 0.0034). The risk model demonstrated good predictive performance (1-/2-/3-year area under the curve = 0.72/0.76/0.83), and its performance was externally validated in an independent cohort. Notably, risk stratification was associated with distinct immune infiltration patterns (including natural killer cells and regulatory T cells), focal adhesion pathway enrichment, and differential drug sensitivity. A nomogram incorporating the risk score, race, and lymphovascular invasion exhibited improved prognostic stratification.

**Conclusion:**

This study presents the first MAM-related prognostic signature specifically tailored to CSCC, filling a gap in existing prognostic models that did not consider the role of MAM. The four-gene risk model offers a robust tool for patient stratification, with potential implications for guiding personalized immunotherapy and targeted therapy. These findings provide a novel framework for incorporating metabolic perspectives into CSCC prognosis and warrant further clinical validation.

## 1 Introduction

Cervical squamous cell carcinoma (CSCC), which affects cervical squamous epithelial cells, comprises approximately 70% of all cervical cancers. The 5-year survival rate is approximately 66%, with more than 30% risk of coming back when it gets worse (1). Standard treatments such as surgery, radiotherapy, and chemotherapy do not adequately prevent tumor recurrence and metastasis (2). Additionally, the absence of specific early symptoms and effective metabolism-related genetic markers often delays diagnosis and treatment. Consequently, investigating the genetic factors associated with metabolism and their involvement in radiotherapy resistance holds significant importance for improving therapeutic outcomes in patients with locally advanced CSCC (3, 4).

Manganese, an essential trace element for the human body, plays a critical role in maintaining physiological homeostasis. It serves as a cofactor for numerous key enzymes involved in antioxidant defense (*e.g.*, manganese superoxide dismutase), energy metabolism, and neurological regulation, while simultaneously modulating immune responses *via* signaling pathways such as the cGAS–STING pathway (5, 6). Recent studies demonstrated that dysregulated manganese metabolism (MAM) is closely associated with tumorigenesis and progression. Manganese affects malignant transformation by modulating oxidative stress and DNA repair (7, 8), and manganese ions enhance antitumor immunity within the tumor microenvironment, demonstrating potential as an immunotherapeutic sensitizer (9). Consequently, aberrant expression of MAM-related genes (MRGs) might contribute to tumor initiation, progression, and therapy resistance through various mechanisms.

MRGs do not represent a functionally homogenous gene set. Broadly, they participate in a wide spectrum of biological processes, ranging from manganese ion transport and storage and regulation of manganese-dependent enzyme expression to various downstream functions influenced by manganese signaling, including antioxidation, DNA repair, and metabolic reprogramming (10). Although some genes might not be exclusively manganese-specific, their functions could be indirectly modulated by manganese ion concentrations or manganese-associated signaling pathways. To date, systematic investigations of MRGs in CSCC remain limited, and the potential of these genes as prognostic biomarkers or therapeutic targets has not been fully explored. Building on previous literature, this study evaluated 1,399 genes potentially linked to MAM (11). By integrating public database analyses with experimental validation, we comprehensively evaluated the expression patterns and prognostic value of MRGs in CSCC; constructed a risk prediction model; and elucidated the roles of these genes on the tumor immune microenvironment, drug sensitivity, and molecular regulatory networks. These findings might provide novel insights and a theoretical foundation for prognostic assessment and individualized therapy in patients with CSCC.

## 2 Materials and methods

### 2.1 Data acquisition

The Cancer Genome Atlas (TCGA)-CSCC cohort was obtained from the University of California, Santa Cruz Xena database (http://xena.ucsc.edu/, accessed on September 15, 2024) and used as training set 1. This dataset comprised 136 CSCC samples. After data download, Ensembl IDs were converted into gene symbols. The original log_2_-transformed FPKM values were converted back to raw counts for subsequent analysis and then re-log_2_–transformed. The GSE52903 dataset was obtained from the Gene Expression Omnibus (GEO) database (http://www.ncbi.nlm.nih.gov/geo/) and used as training set 2. This dataset contained 51 CSCC samples and 17 normal tissue samples (platform: GPL6244). The GSE44001 dataset, comprising 300 CSCC samples with survival information (platform: GPL14951), was also obtained from the GEO database and used as an independent validation set. Both GSE52903 and GSE44001 contained Affymetrix microarray data, and default robust multiarray average normalization, including background correction, quantile normalization, and probe set summarization, had already been performed by the GEO database. Based on this, the probe annotations were cleaned, gene symbols were retained, control/invalid annotations were removed, and duplicate genes were deduplicated. Additionally, disease-free survival (DFS) status and time (converted into days) were extracted from the clinical phenotype data in GSE44001, and samples with incomplete survival information were excluded from subsequent analysis. Furthermore, MRGs were identified on the basis of a 2024 study by Han et al., who compiled 1,399 MRGs by integrating the GeneCards database. This study used this source to obtain MRGs for subsequent analysis (11), as detailed in **Supplementary Table 1**.

### 2.2 Differential expression analysis

The limma package [v.3.54.0 (12)] was used to analyze differentially expressed genes (DEGs) between the tumor and normal groups in the GSE52903 dataset using screening thresholds of |log_2_ fold change (log_2_FC)| > 0.5 and adj. p < 0.05. Specifically, the ggplot2 [v.3.5.1 (13)] and pheatmap packages [v.1.0.12 (14)] were employed to generate volcano plots and heatmaps, respectively, to visualize the results and expression patterns of the significant DEGs. Subsequently, weighted gene co-expression network analysis (WGCNA) was performed using the WGCNA package [v.1.7.1 (15)]. First, hierarchical clustering was conducted to remove sample outliers and determine the optimal soft threshold (β) to ensure that the network conformed to a scale-free topology. β was selected using the pickSoftThreshold() function over the range of 1–20 using the criterion of signed R^2^ ≥ 0.9, and β = 12 was ultimately chosen. Next, the gene adjacency matrix, similarity matrix, and dissimilarity coefficient were calculated sequentially, and a hierarchical clustering tree of genes was constructed with a minimum module size of 10 and a tree cutting height of 0.25. Finally, the plotDendroAndColors function was employed to calculate and visualize the correlation coefficients (cor) between modules and phenotypes. Modules with |cor| > 0.3 and p < 0.05 were selected, and the two modules most strongly correlated with the phenotype (including positive and negative correlations) were identified as key modules. The genes that exhibited significant correlations with CSCC in these two modules were defined as key module genes.

### 2.3 Candidate gene identification and analysis

Using ggvenn [v.0.1.9 (16)], DEGs were intersected with key module genes and MRGs to identify candidate genes. Subsequently, Gene Ontology (GO) and Kyoto Encyclopedia of Genes and Genomes (KEGG) enrichment analyses were conducted using the ClusterProfiler package [v.4.7.1.003 (17)] with a threshold of p < 0.05. GO comprises biological process (BP), cellular component (CC), and molecular function (MF) terms. The candidate genes were imported into the STRING (http://www.string-db.org/) search tool, and a protein–protein interaction (PPI) network was constructed using Cytoscape software [v.3.6.1 (18)] with confidence score ≥ 0.4.

### 2.4 Screening key genes and risk model creation

To identify additional candidate genes related to DFS and prognosis, univariate Cox regression analysis (hazard ratio [HR] ≠ 1, p < 0.05) and the proportional hazards (PH) assumption test (p > 0.05) were performed on all genes in the TCGA-CSCC dataset using the survival package [v.3.5-3 (19)] and the coxph function to screen for prognostic genes. Subsequently, least absolute shrinkage and selection operator (LASSO) regression was performed on the identified prognostic genes using the glmnet package [v.4.1-4 (20)] with 10-fold cross-validation. Genes not shrunk to zero by lambda.min were considered key genes. To investigate the association between the key genes and the manganese homeostasis regulatory network, Spearman’s correlation analysis was performed using the TCGA-CSCC cohort alongside six representative manganese transport and homeostasis regulatory genes, including *SLC39A8*, *SLC39A14*, *SLC30A10*, *ATP13A2*, *TMEM165*, and *SLC11A2* (21–25). Statistical significance was denoted by |cor| > 0.3 and p < 0.05.

A risk model was created using multivariate Cox regression with the following formula (Equation 1):

(1) Risk score =∑coef(genei)×expr(genei)

where coef is the regression coefficient and expr is the gene expression level. After screening the key genes and constructing the risk model, the optimal cutoff of the continuous risk score was calculated using the surv_cutpoint() function from the survminer package [v.0.4.9 (26)] based on the risk scores of each sample in the TCGA-CSCC cohort. This function traversed all possible cut points, evaluated the survival differences between the resulting groups using the log-rank test, and selected the value that yielded the most significant difference in survival curves between the two groups (*i.e.*, the smallest p-value) while ensuring that the size of each group was no less than 30% of the total sample size (minprop = 0.3) as the optimal cutoff. Based on this cutoff, patients in the TCGA-CSCC cohort were divided into high-risk (HRG) and low-risk groups (LRG). Subsequently, Kaplan–Meier (KM) survival curves were plotted using the survminer package and the log-rank test, and the grouping effect was visualized using risk score distribution and survival status plots. Time-dependent receiver operating characteristic (ROC) curves for 1-, 2-, and 3-year survival were plotted using the survivalROC package [v.1.0.3.1 (27)] to evaluate the predictive performance of the risk model. Finally, the same analysis was performed on the GSE44001 dataset to validate the risk model’s predictive performance.

### 2.5 Independent prognostic analysis and nomogram

The DFS prognosis risk model for patients with CSCC included clinical features such as risk scores, age, and population ancestry. First, we tested the PH assumption (p > 0.05) and performed univariate Cox regression analysis (p < 0.05). Next, we performed multivariate Cox analysis (p < 0.05) using the survival package (v.3.5-3) to screen for independent prognostic factors. Based on these factors, a nomogram was created to predict 1-, 2-, and 3-year survival using the rms package [v.6.5-0 (28)]. Calibration curves were drawn using the rms package, and ROC curves were drawn using the timeROC package [v.0.4 (29)] to validate the nomogram. Additionally, the ggDCA package [v.1.2, https://rdrr.io/cran/ggDCA/) was used to generate decision curve analysis (DCA) curves to evaluate the model’s clinical effectiveness.

### 2.6 Gene set enrichment and immunological analyses

Gene set enrichment analysis (GSEA) was conducted to investigate the biological functions of the proteins encoded by key genes during CSCC progression. Differential expression between the HRG and LRG was assessed using the DESeq2 package [v.1.38.0 (30)], and DEGS were ranked by log_2_FC in descending order. GSEA was performed using “c2.cp.kegg.v.2023.1.Hs.symbols,” which was retrieved from the GSEA database (https://www.gsea-msigdb.org/gsea/msigdb/index.jsp) as the background set (adj. p < 0.05). For immune cell infiltration, the ssGSEA algorithm was used to obtain 28 immune cell scores in the HRG and LRG (31). A heatmap of the 28 immune cell types was plotted using the pheatmap package (v.1.0.12), and statistical analysis was performed using the Wilcoxon test (p < 0.05). Moreover, the psych package (v.2.2.9) was used to analyze the correlations among differential immune cells, whereas the ggcor package [v.0.9.8.1 (32)] was used to analyze the correlations between key genes and differential immune cells. Spearman’s correlation analysis was performed with the parameters |cor| > 0.3 and p < 0.05.

### 2.7 Somatic mutation and drug sensitivity analyses

To more deeply evaluate the tumor pathogenesis of CSCC, the epigenetic mutation status of patients in the HRG and LRG was assessed using the oncoplot function in the maftools package [v.2.14.0 (33)], and the top 20 mutated genes were visualized. Subsequently, we conducted pharmacogenomic analysis in the Genomics of Drug Sensitivity in Cancer (GDSC) database (https://www.cancerrxgene.org/) using pRRophetic [v.0.5 (34)] to determine the response to chemotherapy in each risk group. The Wilcoxon test (p < 0.05) was applied to determine the half-maximal inhibitory concentration (IC_50_) of chemotherapeutics and targeted therapies, and the IC_50_s of the five drugs with the most significant differences in efficacy between the groups were listed.

### 2.8 Clinical correlation and molecular regulatory network

To illustrate the relationships among clinical features, risk scores, and key genes, we used the Wilcoxon and Kruskal–Wallis tests (p < 0.05). Subsequently, transcription factors (TFs) associated with the key genes were predicted using the NetworkAnalyst database (http://www.networkanalyst.ca), and a TF–mRNA regulatory network was drawn using Cytoscape (v.3.6.1).

### 2.9 RNA extraction, cDNA synthesis, real-time quantitative polymerase chain reaction, and data analysis

The human immortalized cervical squamous epithelial cell line Ect1/E6E7 (Cat. No.: JNO-H0285) was purchased from Guangzhou Jennio Biotech. The human cervical cancer epithelial cell line CaSki (intestinal metastasis-derived, Cat. No.: CL-0048) and human CSCC cell line SiHa (Cat. No.: CL-0210) were purchased from Procell Life Science & Technology. Both CaSki and SiHa cells were verified by short tandem repeat profiling to ensure cell line authenticity, and each cell line was cultured at a density of 1 × 10^7^ cells per T25 flask. All cells were cultured under sterile conditions following the manufacturer’s recommended protocols. RNA was extracted from CaSki, SiHa, and Ect1 cells using RNA Simple. A Total RNA Extraction Kit (DP419; Tiangong Biotech, Beijing, China) was used according to the supplied protocol. RNA was quantified (260/280) and confirmed by agarose gel electrophoresis (1%), and its integrity was evaluated by agarose gel electrophoresis (1% agarose; visible 28S/18S rRNA bands). cDNA was generated using 800 ng of total RNA and HiScript III All-in-one RT SuperMix Perfect for qPCR (R333-01, Vazyme Biotech, Nanjing, China) using a protocol of 37 °C for 15 min and 85 °C for 5 s (inactivation of reverse transcriptase). Diluted cDNA was stored at −20 °C for further application. RT-qPCR was performed on a Roche LightCycler^®^ 480 II system with ChamQ Blue Universal SYBR qPCR Master Mix (Q312-03, Vazyme Biotech). The 10-µL reaction mixture contained 5.0 µL of 2× Master Mix, 0.3 µL of the forward/reverse primers (10 µM), 4.4 µL of the cDNA template (10 ng equivalent), and a suitable volume of nuclease-free water to a final volume of 10 µL. Amplification was performed at 95 °C for 30 s, denaturation was performed at 95 °C for 10 s; annealing/extension was performed at 60 °C for 30 s; and a melting curve was created to check the product specificity. Target gene expression was normalized to that of the endogenous control *GAPDH*, and relative expression was calculated according to the 2^−ΔΔCt^-method (35). The primer sequences used for RT-qPCR are listed in **Supplementary Table 2**.

### 2.10 Western blotting of key MRG proteins in CSCC and healthy cells

Western blotting was performed on proteins encoded by the key genes to validate MRG expression in CaSki, SiHa, and Ect1 cells. Logarithmic-phase cells were harvested, washed once with precooled PBS (pH 7.4), lysed in RIPA buffer (1% PMSF + 1% protease inhibitor; Beyotime) on ice for 30 min, and centrifuged (12,000 × *g*, 4 °C, 15 min), and the supernatants were collected as total protein extracts. Ponceau S staining confirmed protein integrity, and protein concentrations were standardized using an HRP-conjugated GAPDH antibody (1:10,000, Proteintech). Using a 5% stacking gel and 8%/10%/12% separating gels according to the target protein molecular weight, SDS–PAGE was performed (80 V, 30 min; 120 V, 90–120 min), followed by wet transfer to PVDF membranes (0.22 μm, Millipore) at 300 mA (60–90 min), with transfer confirmed by Ponceau S staining. Membranes were blocked with 5% skimmed milk/TBST at room temperature for 1 h and then incubated with primary antibodies against PARP1 (1:1,000, Abiowell, AWA10084), GALNTL6 (1:1,000, Abiowell, AWA49318), SLC20A1 (1:1,000, Abiowell, AWA67473), TAGLN (1:1,000, Abiowell, AWA10249), and GAPDH (1:1,000, Proteintech, 60004-1-Ig) overnight at 4 °C. Membranes were incubated with HRP-conjugated secondary antibodies at room temperature for 1 h (1:10,000, Proteintech). Bands were visualized *via* exposure to ECL substrate (Beyotime) on the Tanon 5,200 imaging system. ImageJ was used to quantify gray values. Experiments were performed in ≥3 biological replicates, and R software (v.4.2.2) was used to analyze the data using the Wilcoxon and Kruskal–Wallis tests, with p < 0.05 denoting significance.

### 2.11 Immunofluorescence staining of the subcellular localization and expression of PARP1/TAGLN

Immunofluorescence staining was conducted to detect the subcellular localization and relative expression of PARP1 and TAGLN. PARP1 expression was assessed in three cell lines (CaSki, SiHa, and Ect1/E6E7), whereas TAGLN expression was evaluated in CaSki and Ect1 cells. Sterile glass coverslips (Φ12 mm) were placed in 24-well plates, and 5 × 10^4^ cells/well were seeded and cultured at 37 °C with 5% CO_2_ for 24 h until cell confluency reached 60%–70%. The culture medium was discarded, cells were washed three times with precooled PBS (pH 7.4, 5 min per wash), fixed with 4% paraformaldehyde (Beyotime) at room temperature for 15 min, and washed three times with PBS. Fixed cells were permeabilized with 0.1% Triton X-100 in PBS at room temperature for 10 min, washed three times with PBS, and blocked with 5% BSA in PBS for 1 h at room temperature to block nonspecific antibody binding.

Primary antibodies against PARP1 (AWA10084) and TAGLN (AWA10249, Abiowell) were diluted to 1:50 in 1% BSA/PBS, and cells were covered with coverslips and incubated with the antibodies in a humidified dark box at 4 °C for 12 h. All subsequent incubation and washing steps were completed under dark conditions to prevent fluorescence quenching. After three PBS washes (5 min each), cells were incubated with Alexa Fluor™ 488-Goat Anti-Rabbit IgG (H + L) secondary antibody (1:500, Cat No. A-11008, Thermo Fisher Scientific) at room temperature for 1 h. Cell nuclei were counterstained with DAPI working solution (1:1,000, Cat No. D1306, Thermo Fisher Scientific) for 5 min at room temperature and washed three times with PBS. Coverslips were mounted cell-side down onto glass slides with anti-fade mounting medium (Beyotime), and slide edges were sealed with nail polish to avoid drying.

Fluorescent images were captured using a Zeiss LSM980 laser-scanning confocal microscope with identical exposure parameters for all samples, namely excitation/emission wavelengths of 488/525 nm for green signals of PARP1 and TAGLN and 350/460 nm for blue DAPI nuclear signals. For quantitative analysis, ImageJ (v.1.8.0) was used to calculate the mean fluorescence intensity of target proteins, which was normalized to the total number of nuclei in each field of view to assess relative expression. At least five random nonoverlapping fields were captured per coverslip, and all immunofluorescence experiments were repeated independently for three biological replicates. Statistical comparisons among Ect1, SiHa, and CaSki cells were performed using the Kruskal–Wallis test followed by a pairwise Wilcoxon’s test in R (v.4.2.2), and p < 0.05 indicated statistical significance.

### 2.12 Data analysis

ImageJ (v.1.8.0) was used to measure the mean fluorescence intensity of the target protein per cell and normalize the nuclear counts in the same field of view to determine relative expression. For each coverslip, ≥5 random fields of view were selected, and all experiments were performed in three independent biological replicates. Statistical analyses (Wilcoxon, Kruskal–Wallis) were performed in R (v.4.2.2; p < 0.05).

## 3 Results

### 3.1 Identification of DEGs and key module genes in CSCC

In total, 2,777 DEGs were identified in the training set. Among them, 1,177 genes were upregulated and 1,600 genes were downregulated in the CSCC group compared with their expression in the control group (**Figures 1A, B**). WGCNA verified the absence of outliers in the samples (**Figure 1C**). The optimal β was determined to be 12 (R^2^ > 0.9; **Figure 1D**). The network was scale-free, and 32 modules were identified (**Figure 1E**). Correlation analysis demonstrated that the MEgreen and MEred modules had the strongest positive (r = 0.801) and negative correlations (r = −0.839) with the tumor group (both p < 0.0001), respectively (**Figure 1F**). Thus, these two modules were considered closely related to CSCC. The genes from these two modules were integrated to obtain 2,254 key module genes.

**Figure 1:**
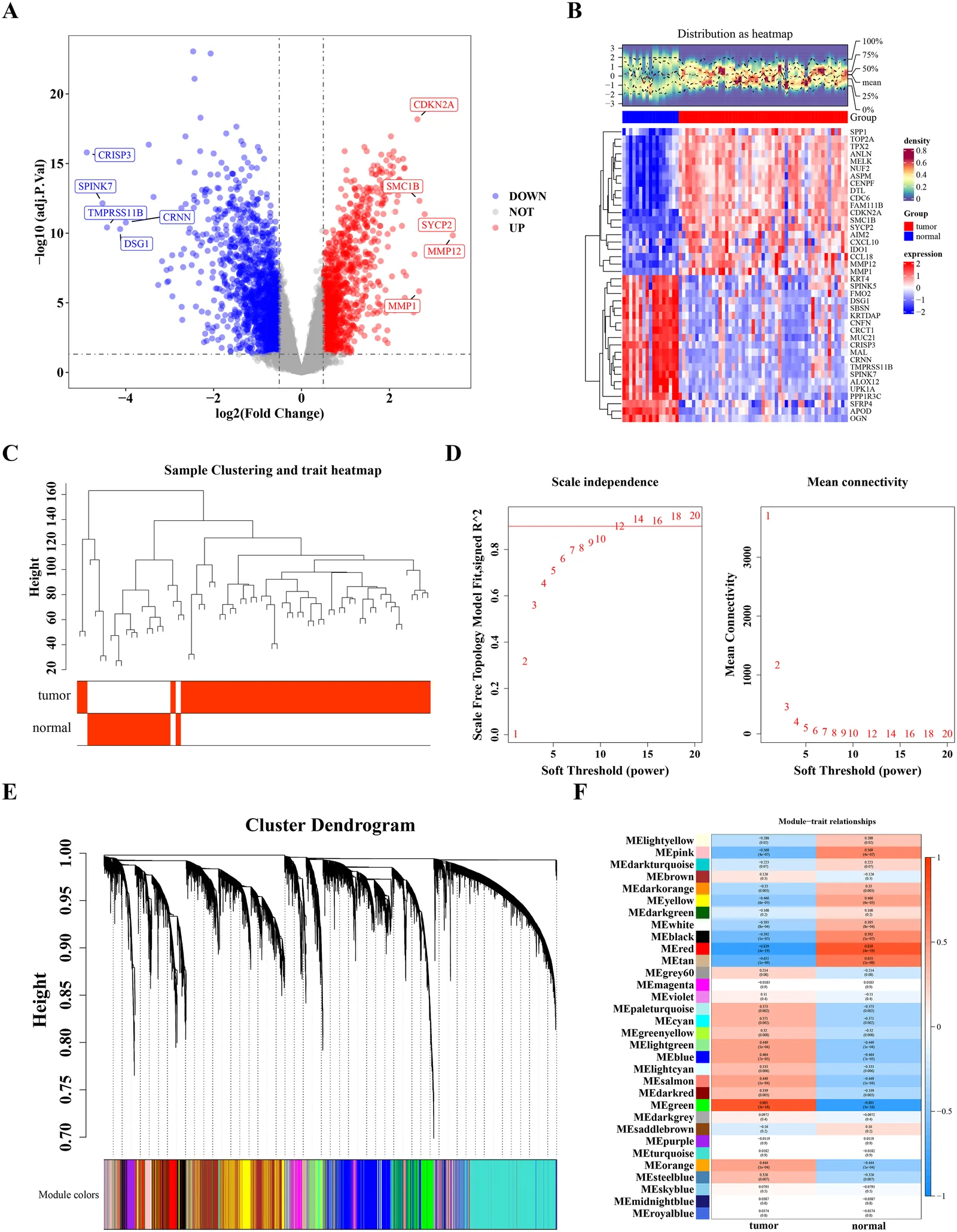
Identification of DEGs and key modules using WGCNA in CSCC (DEGs: Wilcoxon rank sum test; WGCNA: Spearman correlation analysis). **(A)** Volcano plot of DEGs between tumor (n = 51) and normal (n = 17) tissues in GSE52903. X-axis: log_2_FC; Y-axis: -log_10_ (adjusted P-value). DEGs were defined as |log_2_FC|>0.5 and adj. p < 0.05, with key genes labeled. **(B)** Heatmap of the top 40 DEGs (20 upregulated, 20 downregulated) in GSE52903. The color gradient indicates low (blue) to high (red) expression; samples are grouped by tumor/normal. **(C)** Sample clustering and trait heatmap for WGCNA. No outliers were detected; the color bar denotes the sample groups. **(D)** Optimal soft threshold (β = 12) determination for WGCNA. Left: scale-free topology model fit (R² > 0.9); right: mean connectivity. **(E)** Gene cluster dendrogram and module colors. Genes were clustered into 32 modules (gray module excluded). **(F)** Module–trait correlation heatmap. Numbers in cells represent correlation coefficients and P-values (parentheses). MEgreen (cor = 0.801, p < 3e−16) and MEred (cor = −0.839, p < 4e−19) were identified as key modules. CSCC, cervical squamous cell carcinoma; DEGs, differentially expressed genes; WGCNA, weighted gene co-expression network analysis.

### 3.2 Candidate gene identification and functional analysis in CSCC

Intersection of the 2,777 DEGs, 1,399 MRGs, and 2,254 key module genes yielded 116 genes (**Supplementary Table 3**), which were considered candidate genes for the subsequent analysis (**Figure 2A**).GO analysis revealed 1,491 annotated entries, including 1,335 BP, 47 CC, and 109 MF terms, mainly comprising DNA replication and regeneration, replisome, and nuclear replication fork pathways (**Figure 2B**). KEGG analysis highlighted 96 enriched functional pathways, including DNA replication and focal adhesion pathways (**Figure 2C**). The PPI network featured 116 nodes, and *JUN*, *PTEN*, and *CDK2* displayed many interactions with other genes (**Figure 2D**).

**Figure 2:**
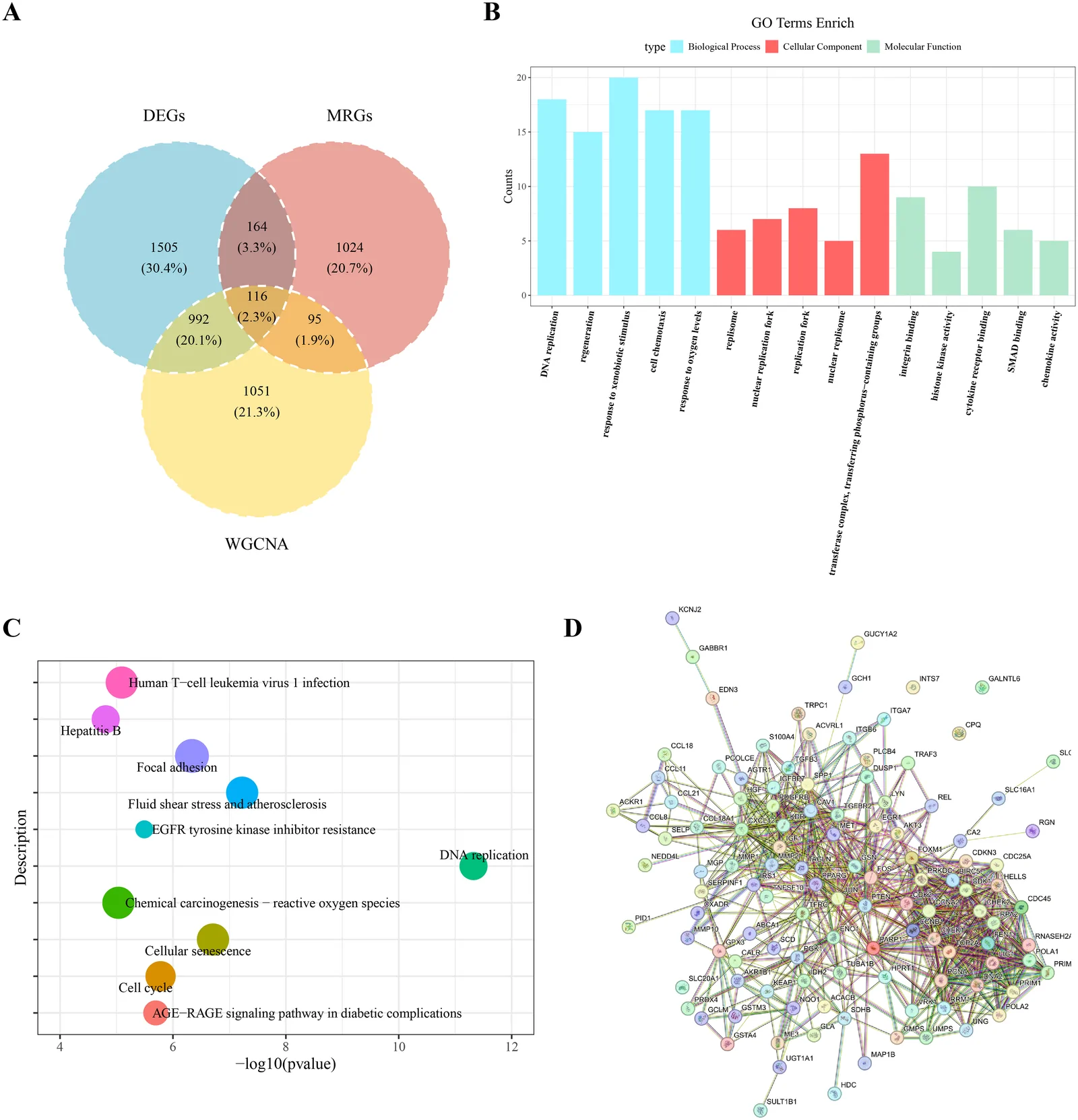
Identification of candidate genes and their functional characteristics. **(A)** Venn diagram showing the intersection of DEGs, MRGs, and WGCNA key module genes. The overlapping area (116 genes) represents candidate genes for subsequent analysis, with each circle labeled with gene counts and percentages. **(B)** GO enrichment analysis of 116 candidate genes. The plot shows the top-enriched terms in the BP, CC, and MF categories, with the y-axis indicating term names and the x-axis indicating gene counts. **(C)** KEGG enrichment analysis of 116 candidate genes. The x-axis represents –log_10_(p-value), and the y-axis lists the top-enriched pathways (e.g., DNA replication, focal adhesion) (GO and KEGG enrichment analysis: hypergeometric test). **(D)** PPI network of candidate genes. Nodes represent genes, edges represent interactions (confidence ≥ 0.4), with core genes (JUN, PTEN, and CDK2) and key MRGs (PARP1, SLC20A1, GALNTL6, and TAGLN) highlighted. DEGs, differentially expressed genes; MRGs, MAM-related genes; WGCNA, weighted gene co-expression network analysis; GO, Gene Ontology; KEGG, Kyoto Encyclopedia of Genes and Genomes; PPI, protein–protein interaction.

### 3.3 Establishment and validation of prognostic models in CSCC

Univariate Cox regression analysis and the PH assumption test identified nine prognosis-related genes (**Figure 3A**). We used LASSO regression analysis (lambda. min = 0.008491522) and multivariate Cox regression analysis to perform further screening, which revealed four key genes (*PARP1*, *SLC20A1*, *GALNTL6*, and *TAGLN*; **Figure 3B**). Spearman’s correlation analysis indicated that *TAGLN* (cor = 0.35, p < 0.001) and *SLC20A1* (cor = 0.39, p < 0.001) were significantly positively correlated with *SLC39A14*, suggesting that *TAGLN* and *SLC20A1* might exhibit transcriptional coexpression with the manganese uptake pathway (**Supplementary Figure 1**). The remaining key genes also displayed positive correlations with the aforementioned manganese regulatory genes, although these correlations did not reach statistical significance. The risk model was built on the TCGA-CSCC dataset as follows: risk score = 2.2294 × *PARP1* + 1.5085 × *SLC20A1* + 1.7624 × *GALNTL6* + 0.7970 × *TAGLN*. Using −0.4920046 as the risk score threshold, TCGA-CSCC samples were divided into the HRG (n = 87) and LRG (n = 49). Risk distribution and survival status plots demonstrated that the HRG had a higher risk score and a shorter survival time (**Figure 3C**). KM survival curves also revealed that patients in the HRG group had a lower survival probability (p = 0.0034; **Figure 3D**). The areas under the curve (AUCs) for 1-, 2-, and 3-year survival according to the risk model all exceeded 0.7, indicating that the risk model had good ability to predict the survival of patients with CSCC (**Figure 3E**). Next, the validation set samples were stratified by the risk score threshold of 68.15301 (HRG, n = 90; LRG, n = 210; **Figure 3F**). The KM survival curve also indicated that patients in the HRG had a reduced probability of survival. The AUCs of the risk model for predicting 1-, 2-, and 3-year survival exceeded 0.6, indicating moderate predictive utility, which, to some extent, demonstrated the validity of the risk model.

**Figure 3:**
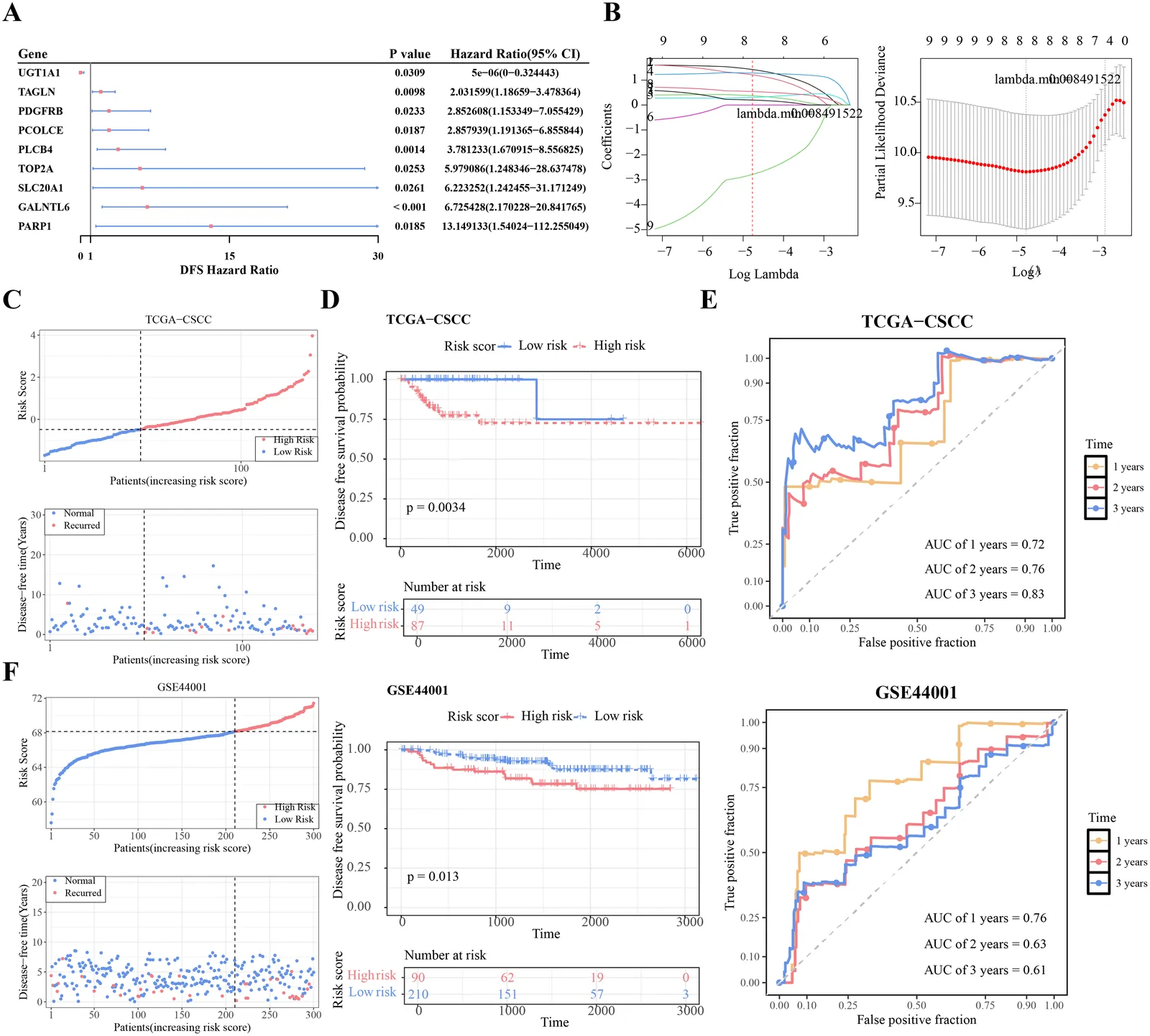
Construction and validation of the prognostic risk model. **(A)** Forest plot of the univariate Cox regression analysis. Genes are colored by risk type (HR > 1: red, risk factors; HR < 1: blue, protective factors), with MRGs marked separately. P < 0.05 was considered statistically significant (univariate Cox analysis: Cox-PH assumption test). **(B)** LASSO regression analysis for key gene screening. Left: coefficient profiles of prognostic genes; right: partial likelihood deviance plot, with lambda.min = 0.008491522 (LASSO: LASSO regression analysis, stepwise multivariate Cox regression analysis). **(C)** Risk score distribution and survival status of patients in the TCGA-CSCC cohort. Patients are ordered by risk score, with high- and low-risk groups divided by the optimal threshold (−0.4920046). **(D)** KM survival curve of TCGA-CSCC. High-risk patients had a significantly worse prognosis (p = 0.0034, log-rank test) (survival analysis: log-rank test). **(E)** ROC curves of the risk model in TCGA-CSCC. AUC values for 1-, 2-, and 3-year DFS were 0.72, 0.76, and 0.83, respectively. **(F)** Validation of the model in GSE44001. Left: KM curve (p = 0.013); right: ROC curves (1-/2-/3-year AUC = 0.76/0.63/0.61). HR, hazard ratio; LASSO, least absolute shrinkage and selection operator; TCGA-CSCC, The Cancer Genome Atlas-cervical squamous cell carcinoma; DFS, disease-free survival; ROC, receiver operating characteristic; AUC, area under the curve.

### 3.4 Nomogram of CSCC based on independent risk factors

The variables were tested in the TCGA-CSCC dataset using univariate Cox regression (p < 0.05) and the PH assumption test (p > 0.05; **Supplementary Table 4**). Multivariate Cox regression revealed that the risk score, white ancestry, and lymphovascular invasion were independent prognostic factors for CSCC (**Figures 4A, B**; **Supplementary Table 5**). Subsequently, a nomogram was established using these independent prognostic factors to predict the 1-, 2-, and 3-year survival of patients with CSCC (**Figure 4C**). The calibration curve was extremely close to the reference line (**Figure 4D**). ROC curve analysis illustrated that the AUCs of the nomogram for 1-, 2-, and 3-year survival were >0.7 (**Figure 4E**). The DCA results indicated that the benefit of the nomogram model was much higher than the oblique and horizontal lines, and the nomogram model had good performance (**Figure 4F**). These findings provide a reference for the clinical prognosis prediction of patients with CSCC.

**Figure 4:**
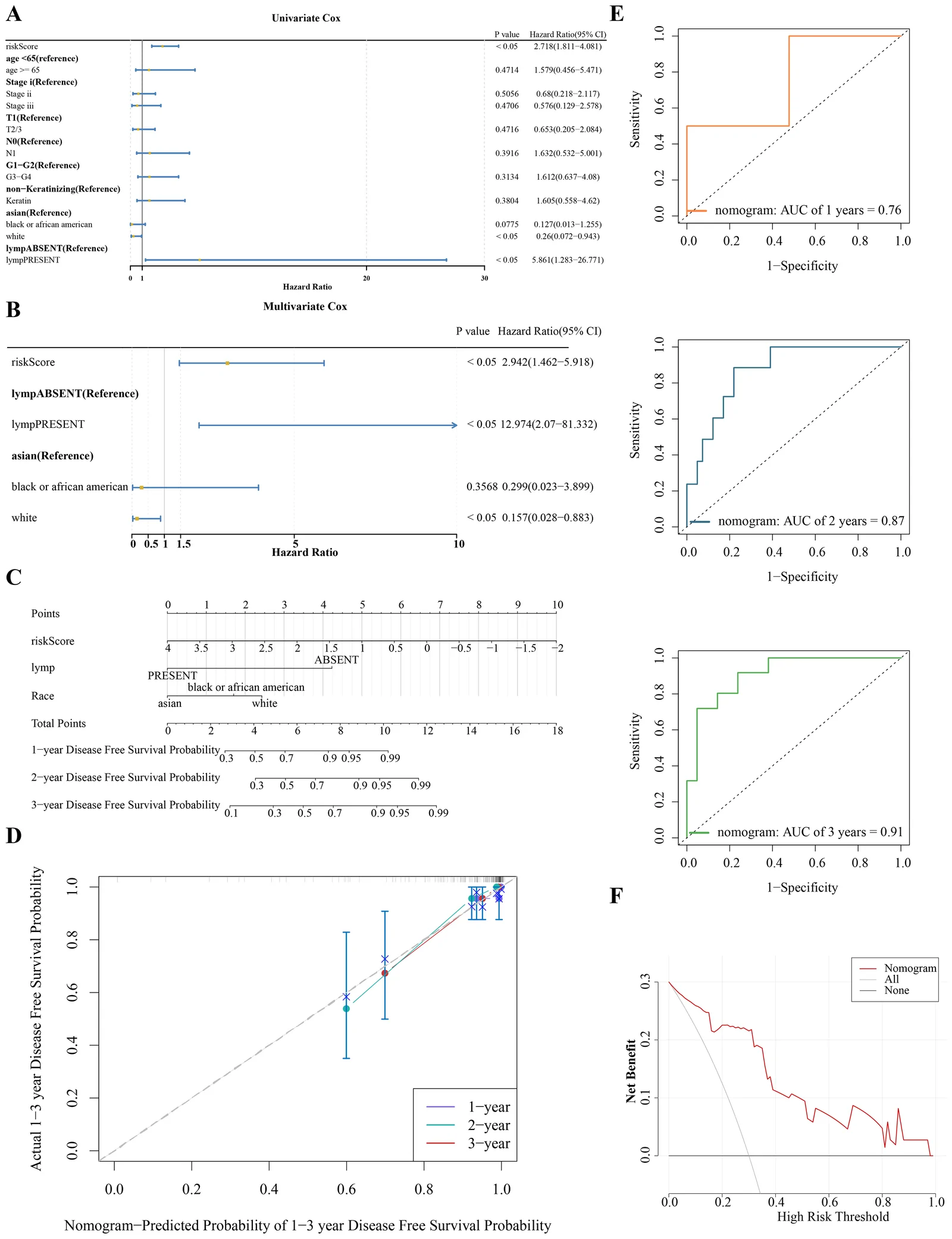
Independent prognostic analysis and nomogram construction. **(A)** Forest plot of univariate Cox regression analysis. Risk score, population ancestry, and lymphovascular invasion were significantly associated with prognosis (p < 0.05). **(B)** Forest plot of multivariate Cox regression analysis. Risk score (HR = 2.942, p < 0.05), white ancestry (HR = 0.157, p < 0.05), and lymphovascular invasion (HR = 12.974, p < 0.05) were identified as independent prognostic factors. **(C)** Nomogram for predicting 1-, 2-, and 3-year DFS of patients with CSCC, integrating independent prognostic factors. **(D)** Calibration curve of the nomogram. The predicted probabilities were highly consistent with actual survival outcomes (nomogram calibration curve: consistency test). **(E)** ROC curves of the nomogram. AUC values for 1-, 2-, and 3-year DFS were 0.76, 0.87, and 0.91, respectively. **(F)** Decision curve analysis of the nomogram. The model showed a higher net clinical benefit than “All” or “None” strategies. DFS, disease-free survival; CSCC, cervical squamous cell carcinoma; ROC, receiver operating characteristic; AUC, area under the curve; DCA, decision curve analysis.

### 3.5 Prediction of risk models in GSEA and the immune microenvironment

The GSEA results revealed that the HRG and LRG were enriched for focal adhesion, extracellular matrix (ECM)–receptor interaction, complement, and coagulation cascade (**Figure 5A**). To determine the associations of key genes with the immune microenvironment, the difference in immune cell infiltration scores between the two groups was evaluated (**Figure 5B**). The infiltration of four immune cell types (including effector memory CD4^+^ T cells) was notably elevated in the HRG (p < 0.05; **Figure 5C**). Correlation heatmaps demonstrated that regulatory T cells (Tregs) and natural killer (NK) cells had the strongest correlation (r = 0.69, p < 0.05), and *TAGLN* expression had the strongest positive correlation with NK cells (r = 0.54, p < 0.05; **Figures 5D, E**). Comparative GSEA and immune infiltration analyses between the HRG and LRG revealed distinct patterns of pathway enrichment and immune cell abundance associated with the risk model. However, these findings were based on computational inference, and further experimental validation is required.

**Figure 5:**
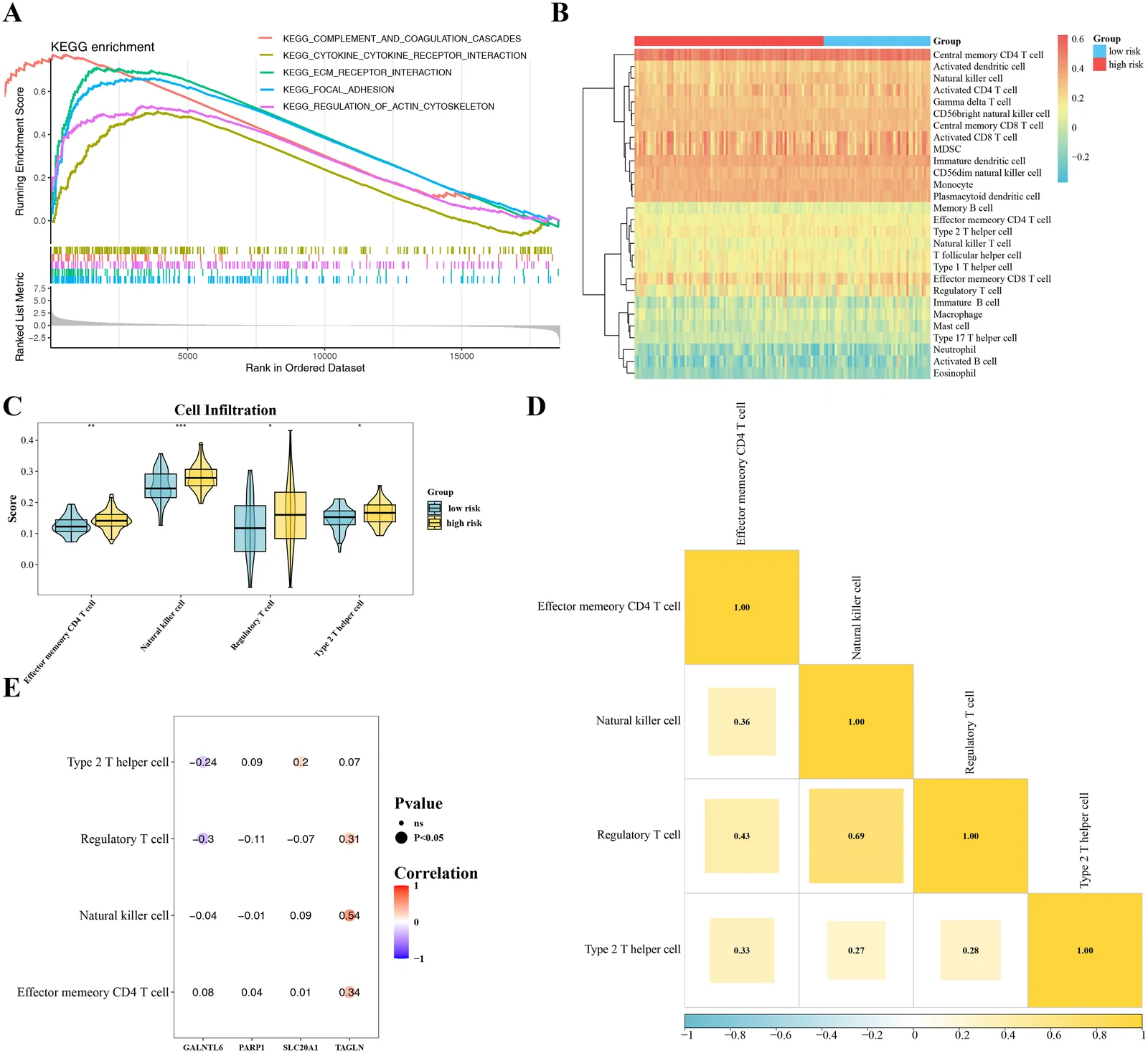
GSEA and tumor immune microenvironment analysis. **(A)** GSEA results comparing high- and low-risk groups in TCGA-CSCC. Top-enriched KEGG pathways included focal adhesion, ECM–receptor interaction, and complement-coagulation cascades (adj. p < 0.05) (GSEA enrichment analysis: permutation test). **(B)** Heatmap of 28 immune cell infiltration scores. Colors indicate relative enrichment levels (blue: low; red: high) in the high- and low-risk groups. **(C)** Differential immune cell infiltration between risk groups. Effector memory CD4+ T cells, NK cells, Treg cells, and Th2 cells were significantly increased in the high-risk group (p < 0.05, Wilcoxon test). **(D)** Correlation heatmap of differential immune cells. Treg cells and NK cells showed the strongest positive correlation (cor = 0.69, p < 0.05). **(E)** Correlation between key genes and differential immune cells. TAGLN expression was positively correlated with NK cells (cor = 0.54, p < 0.05). GSEA, gene set enrichment analysis; TCGA-CSCC, The Cancer Genome Atlas-cervical squamous cell carcinoma; KEGG, Kyoto Encyclopedia of Genes and Genomes; NK cells, natural killer cells; Treg cells, regulatory T cells; Th2 cells, type 2 T helper cells.

### 3.6 Somatic mutations and chemotherapeutic agents in the risk groups

Analysis of somatic mutation data revealed distinct differences in mutation profiles between the HRG and LRG. The genes with the highest mutation frequencies in the HRG were *TTN* (44%) and *PIK3CA* (22%; **Figure 6A**), whereas those in the LRG were *TTN* (36%), *PIK3CA* (34%), and *MUC4* (**Figure 6B**). Additionally, missense mutations represented the most common mutation type in both groups. Subsequently, using drug sensitivity data from the GDSC database, the pRRophetic algorithm was applied to systematically predict the responses of patients within the TCGA-CSCC cohort to chemotherapy and targeted therapy. This analysis covered all available drugs in the database, rather than being limited to drugs already clinically approved for cervical cancer, and explored potential differences in drug sensitivity between the risk subgroups. The results illustrated that patients in the HRG were more sensitive to five drugs, including A.770041 and BX.795, whereas those in the LRG were more sensitive to five other drugs, including BIBW2992 and gefitinib (**Figures 6C, D**). These computational predictions suggested that the risk groups might exhibit potential differences in drug responsiveness, and the risk model could serve as an exploratory basis for selecting candidate drugs in subsequent experimental and clinical studies.

**Figure 6:**
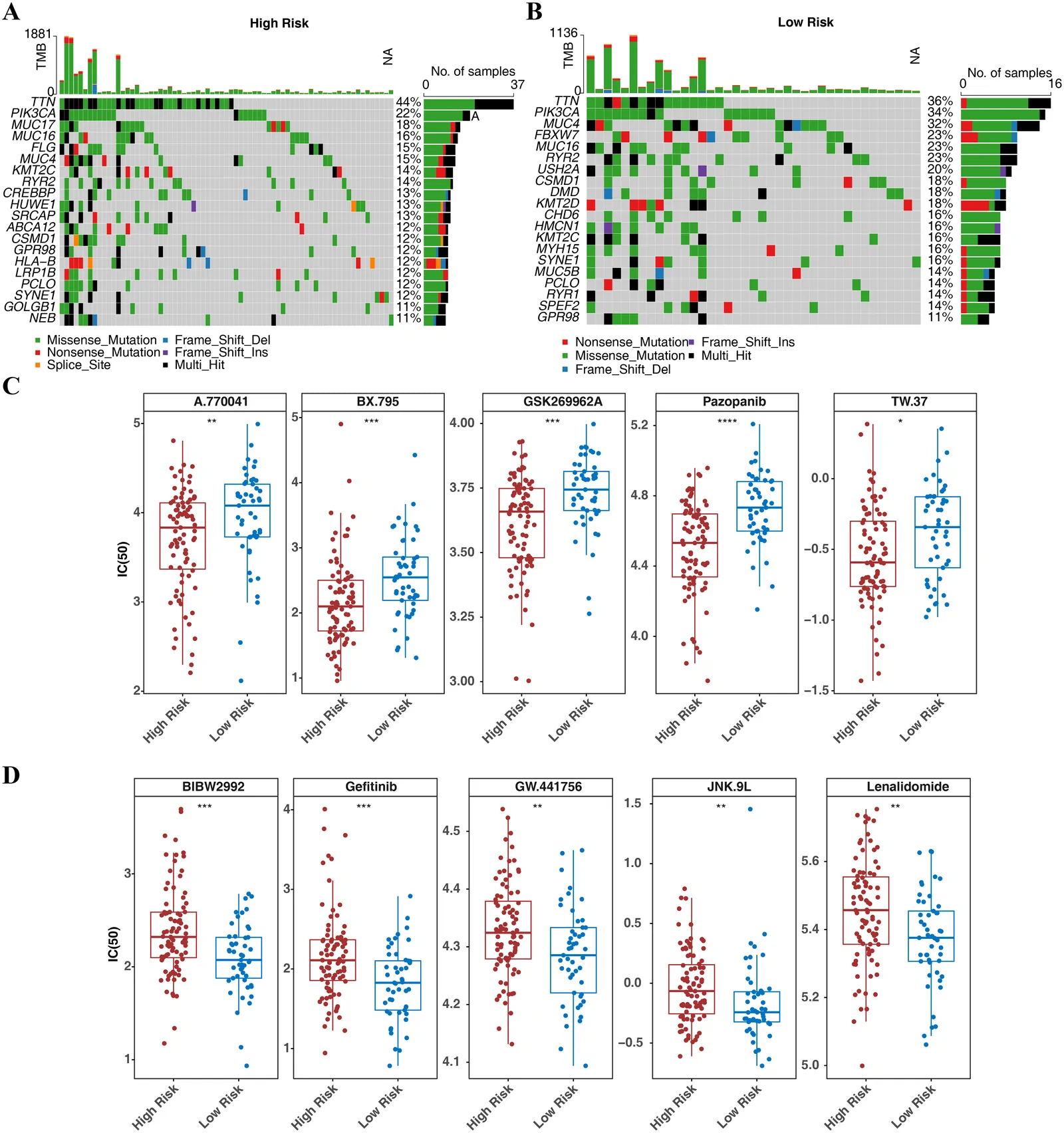
Somatic mutation and drug sensitivity analysis. **(A)** Mutation waterfall plot of the high-risk group (n = 37). The top 20 mutated genes are shown, with TTN (44%) and PIK3CA (22%) being the most frequent. **(B)** Mutation waterfall plot of the low-risk group (n = 16). Top mutated genes also included TTN (36%) and PIK3CA (34%). **(C)** Drug sensitivity in the high-risk group. High-risk patients were more sensitive to A.770041, BX.795, GSK269962A, Pazopanib, and TW.37 (p < 0.05). **(D)** Drug sensitivity in the low-risk group. Low-risk patients were more sensitive to BIBW2992, Gefitinib, GW.441756, JNK.9L, and lenalidomide (p < 0.05) (drug sensitivity analysis: Wilcoxon rank sum test). IC_50_, half-maximal inhibitory concentration.

### 3.7 Biological and regulatory roles of key genes in CSCC

Risk scores differed among different age groups (p < 0.05). Clinical correlation analysis demonstrated that high *SLC20A1* expression was correlated with an age of >65 years (p < 0.05), and high *TAGLN* expression was correlated with grade G3–G4 (p < 0.05). *PARP1* expression was significantly different among different ancestries (p < 0.01; **Figures 7A, B**). In total, 122 TFs were predicted to control the expression of the key genes. TFs highly related to the key genes included *SOX2*, *CEBPB*, *AR*, and *MYCN*. Among these, *SOX2* simultaneously regulated all four key genes (**Figure 7C**). These analyses provide strong support for elucidating the biological regulatory mechanism of CSCC.

**Figure 7:**
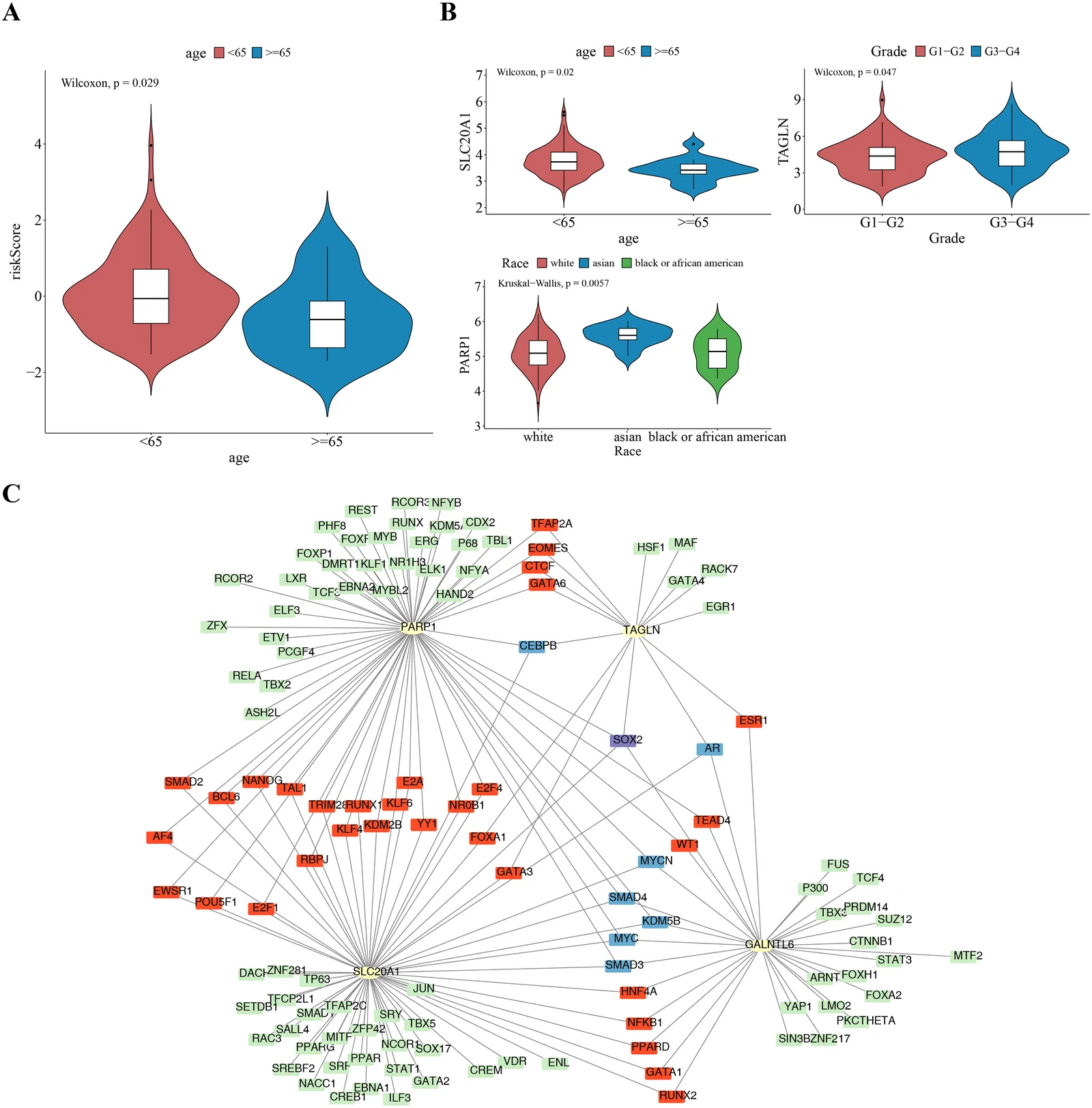
Clinical associations and predicted TF–mRNA regulatory network. **(A)** Distribution of risk scores according to age group (<65 vs ≥65 years); the two groups differed significantly (Wilcoxon rank-sum test, p = 0.029). **(B)** Violin plots showing SLC20A1 expression according to age group (Wilcoxon rank-sum test, p = 0.020), TAGLN expression according to histological grade (G1–G2 vs G3–G4; Wilcoxon rank-sum test, p = 0.047), and PARP1 expression according to race (White, Asian, and Black or African American; Kruskal–Wallis test, p = 0.0057). **(C)** Predicted TF–mRNA regulatory network comprising 122 transcription factors and four key genes (PARP1, SLC20A1, GALNTL6, and TAGLN). SOX2 showed predicted regulatory associations with all four key genes. Nodes represent transcription factors or key genes, and edges indicate predicted regulatory associations. TF, transcription factor; mRNA, messenger RNA.

### 3.8 Validation of the subcellular localization of key genes

Immunofluorescence staining demonstrated that the red fluorescent signal of PARP1 was primarily co-localized with the DAPI-labeled nuclear region, indicating that PARP1 is mainly localized in the cell nucleus. Quantitative analysis of fluorescence intensity indicated that the fluorescence intensity of PARP1 was significantly higher in both CaSki and SiHa cells than in Ect1 cells (p < 0.05; **Figures 8A, B**), confirming that PARP1 protein expression is elevated in the two CSCC cell lines. This result provides experimental validation for the PARP1 expression trend indicated by bioinformatic analysis. The TAGLN fluorescent signal was mainly distributed diffusely in the form of fine granules. The overall distribution pattern of TAGLN was relatively similar between CaSki and Ect1 cells. Quantitative analysis revealed no statistically significant difference in TAGLN fluorescence intensity between CaSki and Ect1 cells (p > 0.05; **Figures 8C, D**). Therefore, under the experimental conditions of this study, the overall fluorescence of TAGLN did not differ between CaSki and Ect1 cells. However, the possibility of subtle differences in the subcellular localization of TAGLN between these cells requires further quantitative verification.

**Figure 8:**
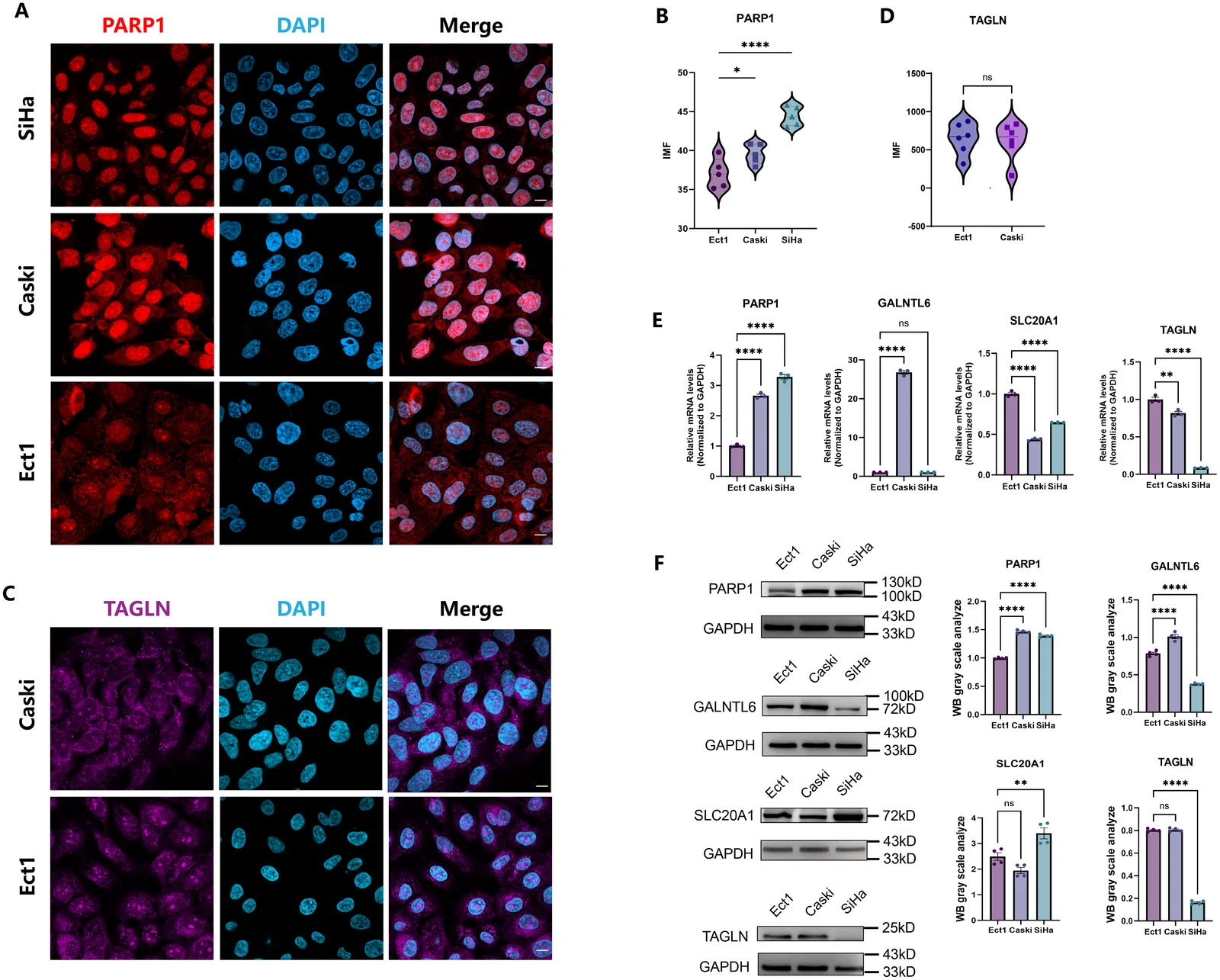
Experimental validation of the expression and subcellular localization of four key manganese metabolism-related genes in cervical squamous cell carcinoma cell lines. **(A)** Immunofluorescence staining of PARP1 (red) in Ect1/E6E7, CaSki, and SiHa cells. Nuclei were counterstained with DAPI (blue). Scale bar = 20 μm. **(B)** Quantitative analysis of the mean fluorescence intensity (MFI) of PARP1 in **(A)**. Data are presented as violin plots with individual data points overlaid. **(C)** Immunofluorescence staining of TAGLN (magenta) in Ect1/E6E7 and CaSki cells. Nuclei were counterstained with DAPI (blue). Scale bar = 20 μm. **(D)** Quantitative analysis of the mean fluorescence intensity (MFI) of TAGLN in **(C)**. **(E)** Relative mRNA expression levels of PARP1, GALNTL6, SLC20A1, and TAGLN in Ect1, CaSki, and SiHa cells determined by RT-qPCR. Expression was normalized to GAPDH using the 2^−ΔΔCt^ method. **(F)** Western blot analysis and quantitative gray-value analysis of PARP1, GALNTL6, SLC20A1, and TAGLN protein expression in Ect1, CaSki, and SiHa cells. GAPDH was used as the loading control. Molecular weight markers (kDa) are indicated on the right of each blot. All experiments were independently repeated at least three times. Statistical comparisons were performed using the Kruskal–Wallis test followed by pairwise Wilcoxon rank-sum tests. p < 0.05, *p < 0.01, **p < 0.001, ***p < 0.0001; ns, not significant.

### 3.9 Validation of key genes at the transcriptional level

RT-qPCR demonstrated that *PARP1* expression was considerably higher in CaSki (2.6-fold, p < 0.0001) and SiHa cells (3.2-fold, p < 0.0001) than in Ect1 cells. *GALNTL6* was strongly upregulated in CaSki cells (27-fold, p < 0.0001), and *SLC20A1* was considerably downregulated in CaSki (0.4-fold, p < 0.0001) and SiHa cells (0.7-fold, p < 0.0001). *TAGLN* was highly expressed in Ect1 cells but considerably downregulated in CaSki (0.8-fold, p < 0.01) and SiHa cells (0.1-fold, p < 0.0001). These results aligned the bioinformatics prediction trends in the TCGA-CSCC and GSE52903 datasets (**Figure 8E**).

### 3.10 Translational validation of key genes

On western blotting, the gray value of the PARP1 (≈113 kDa) protein band was significantly higher in CSCC cells than in Ect1 cells (p < 0.0001). GALNTL6 (≈72 kDa) protein expression was highest in CaSki cells, extremely low in Ect1 and SiHa cells (p < 0.0001). SLC20A1 expression (≈70–75 kDa) was significantly lower in CaSki cells than in Ect1 and SiHa cells, whereas no significant difference was observed between Ect1 and SiHa cells. The molecular weight was higher than the theoretical value (≈43 kDa) because of posttranslational modification. TAGLN expression (≈22 kDa) was significantly higher in Ect1 and CaSki cells than in SiHa cells (p < 0.0001), whereas no significant difference was found between Ect1 and CaSki cells. These results suggest that the protein expression trends of the four key genes were generally consistent with their transcriptional levels, and their differential expression remained stable in CSCC cells, providing a basis for further research on their functional mechanisms (**Figure 8F**).

## 4 Discussion

CSCC is among the most common malignant tumors in women, and it carries an extremely poor prognosis. The efficacy of current therapies is limited by recurrence and metastasis, as well as a lack of specific biomarkers for early diagnosis and intervention. MAM maintains tumor formation and regulates immunological homeostasis. The curative effects, as well as the underlying mechanism by which MRGs influence CSCC, remain unclear (5, 36). We screened prognosis-relevant MRGs by analyzing publicly available bulk RNA-seq data alongside bioinformatic analysis, identifying four key genes. We developed a prognostic risk model and incorporated clinical factors to create a high-performance nomogram to predict survival. Risk stratification was associated with differences in tumor immune infiltration and drug sensitivity. Experimental validation confirmed that these four MRGs were differentially expressed. These results shed light on the regulatory roles of MRGs in the pathophysiology of CSCC and provide tools to address the clinical need for CSCC treatments.

Based on existing literature and data from this study, we investigated the roles of four MRGs (*PARP1*, *SLC20A1*, *GALNTL6*, and *TAGLN*) in the pathophysiology of CSCC, as well as their associations with MAM. PARP1 is a key enzyme involved in DNA repair and apoptosis (37). Recent studies have indicated that manganese ions directly regulate PARP1 function. Sukhanova et al. demonstrated through *in vitro* experiments that Mn^2+^ induces liquid–liquid phase separation of poly(ADP-ribosyl)ated PARP1, forming multimolecular assemblies. This process modulates PARP1’s automodification activity and its interactions with downstream repair proteins (38). Moreover, high-concentration Mn^2+^ exposure induces intracellular oxidative stress, generating excessive reactive oxygen species and leading to oxidative DNA damage (39). Such damage activates the base excision repair (BER) pathway (40). As a critical component of BER, PARP1 is recruited to DNA damage sites, where it synthesizes poly(ADP-ribose) chains in large quantities (41). Furthermore, compared with the findings in normal cervical Ect1 cells, *PARP1* was significantly upregulated in CSCC cell lines (CaSki and SiHa). Its overexpression might promote cancer cell proliferation and resistance to radiotherapy and chemotherapy by enhancing DNA repair (42–44). Based on this evidence, we hypothesize that manganese might contribute to CSCC progression by modulating PARP1 activity.

*SLC20A1* encodes sodium-dependent phosphate transporter 1, which is primarily involved in maintaining cellular phosphate homeostasis (45). Although no direct interaction between SLC20A1 and manganese has been reported, studies have demonstrated that manganese exposure can induce intracellular oxidative stress (46), which influences the expression and function of phosphate transporters (47). Compared with the findings in Ect1 cells, SLC20A1 mRNA expression was decreased in CSCC cell lines, and its protein level was significantly lower in CaSki cells, whereas no significant difference was observed between SiHa and Ect1 cells. We hypothesize that manganese might indirectly modulate *SLC20A1* expression by altering oxidative stress, thereby contributing to metabolic reprogramming in CSCC.

*GALNTL6* encodes the polypeptide N-acetylgalactosaminyltransferase-like 6 (48), which initiates mucin-type O-glycosylation, a process implicated in cancer invasion and metastasis (49). AS manganese ions serve as essential cofactors for glycosyltransferases (50), we postulate that manganese might participate in O-linked glycosylation catalyzed by GALNTL6. Therefore, manganese could influence glycosylation in tumor cells by modulating GALNTL6 enzymatic activity, thus contributing to CSCC progression.

*TAGLN* encodes the tumor suppressor transgelin, an actin-binding protein involved in actin crosslinking and cell migration. Studies have indicated that manganese exposure induces oxidative stress (46), and oxidative stress signaling pathways regulate *TAGLN* expression (51). Additionally, TAGLN expression is lost in the early stages of CSCC development, and at the transcriptional level, its expression was significantly lower in CaSki and SiHa cells than in Ect1 cells. Patients with low *TAGLN* expression exhibit higher risk scores and shorter survival times (52–54). We speculate that manganese might influence *TAGLN* expression *via* oxidative stress regulation, thereby participating in cytoskeletal remodeling and migration control in CSCC.

Furthermore, the prognostic four-gene model demonstrated favorable predictive capability in the training cohort, with AUCs of 0.72, 0.76, and 0.83 for 1-, 2-, and 3-year survival, respectively. Notably, a prognostic model constructed from lipid metabolism-related genes yielded 1-, 3-, and 5-year AUCs of 0.76, 0.75, and 0.68, respectively (55); whereas a model based on anoikis-related genes achieved 1-, 3-, and 5-year AUCs of 0.776, 0.732, and 0.735, respectively (56). Compared with these two existing models, the current model displayed comparable overall predictive performance, with a slight advantage in medium- to long-term (3 years) survival prediction. More importantly, this work is the first to incorporate the perspective of MAM, a relatively underexplored research area, into the prognostic assessment of CSCC, which broadens the research framework for metabolism-related prognostic markers. Nevertheless, this study utilized publicly available datasets, which can introduce certain bias and overfitting risks, and it lacked prospective or large-scale external validation. Future investigations are required to recruit multicenter, independent external validation cohorts and conduct prospective studies to evaluate the generalizability and clinical utility of the proposed model. In addition, integrating the current model with other types of prognostic models can be explored to further improve the robustness of prediction.

Concerning functional enrichment analysis, focal adhesion (57–59), ECM–receptor interactions (60–62), receptor signaling other than ECM–receptor interactions, and cell adhesion/migration (excluding focal adhesion, focusing on motility) were identified as significantly enriched pathways. The mechanisms of CSCC progression remain incompletely understood, and additional putative therapeutic targets might be identified by focusing on these pathways. Specifically, focal adhesion pathways (which are important for tumor cell adhesion, migration, and intercellular signaling) are abnormal in CSCC. The four MRGs disrupt normal signal transmission through focal adhesion pathways, promoting the development of tumors and metastasis (63, 64). Additionally, dysregulation of focal adhesion kinase promotes cell motility and invasiveness, thereby enhancing CSCC aggressiveness. Receptor signaling pathways might allow the four MRGs to regulate oncogenic cascades mediated by EGFR/HER2, and downstream PI3K/AKT and MAPK pathways are important pathways in the pathogenesis of CSCC (52, 53, 65). Furthermore, the involvement of cell adhesion and migration pathways (cancer metastasis is related to the migration and adhesion of cancer cells) suggests that the four MRGs might affect CSCC prognosis by regulating epithelial–mesenchymal transition, which is closely associated with increased invasiveness and poor prognosis in CSCC (66). Additional research is needed to explore the regulatory relationships of the four MRGs with CSCC.

In this study, we observed that the abundance of effector memory CD4^+^ T cells, NK cells, Tregs, and Th2 cells was significantly increased in the HRG based on transcriptomic data, and *TAGLN* expression was positively correlated with NK cell abundance. This presents an apparently paradoxical phenomenon: prognosis was worse in the HRG, but the abundance of multiple immune cell types was elevated in this group. We hypothesize that this phenomenon might be related to the functional status and spatial distribution of immune cells in the tumor microenvironment, which requires further experimental validation. The enrichment of Tregs and Th2 cells in the HRG indicated a potential tendency of immunosuppression. Previous research reported that Tregs can inhibit the functions of CD8^+^ T cells and NK cells by secreting IL-10 and TGF-β (67), whereas Th2 cells promote the M2 polarization of macrophages through the IL-4/IL-13 signaling pathway, thereby attenuating antitumor immune responses (68). In this context, despite the increased numbers of effector memory CD4^+^ T cells and NK cells, their antitumor functions might be suppressed (69, 70). Furthermore, NK cells in the tumor microenvironment often exhibit functional exhaustion or inactivation (71). In this study, the positive correlation between Treg and NK cell abundance (r = 0.69) and the positive correlation between *TAGLN* [an activation marker of cancer-associated fibroblasts (CAFs)] and NK cells (r = 0.54), combined with previous reports that Treg-derived TGF-β inhibits NK cell function (67, 71) and CAFs mediate ECM remodeling to restrict immune cell infiltration into the tumor parenchyma (72, 73), indicate that the NK cell enrichment observed in the HRG might reflect the accumulation of functionally impaired cells rather than enhanced cytotoxic activity. Meanwhile, CAF activation can cause immune cells to be trapped in the stromal region, preventing them from effectively contacting and eliminating tumor cells. In conclusion, the coexistence of elevated immune cell abundance and poor prognosis in the HRG might reflect a state of immune dysregulation. However, immune cell abundance was inferred from bulk transcriptomic data solely based on the ssGSEA algorithm, and neither their functional status nor spatial distribution was experimentally validated. Therefore, the explanation proposed in this study should be regarded as a speculative hypothesis based on existing research rather than a definitive conclusion, and further validation *via* multiplex immunohistochemistry and cell functional experiments is required in subsequent studies.

Using the GDSC database, we analyzed drug sensitivity in the TCGA-CSCC cohort. The results revealed significant differences in drug sensitivity between the risk groups. The HRG exhibited increased sensitivity to pazopanib, a multitarget tyrosine kinase inhibitor that suppresses angiogenesis and tumor proliferation by inhibiting receptors such as VEGFR, PDGFR, and C-KIT, thereby blocking pathways including focal adhesion and ECM–receptor interactions. This finding suggests that angiogenesis-related pathways might represent a potential therapeutic direction worthy of further investigation in the HRG. Contrarily, the LRG exhibited greater predicted sensitivity to EGFR inhibitors such as gefitinib. AS EGFR might be overexpressed or aberrantly activated in CSCC and it is associated with downstream oncogenic pathways such as PI3K/AKT and MAPK, EGFR inhibitors could represent candidate therapeutic modalities warranting further validation in the LRG (74). Additionally, other drugs with potential efficacy in the HRG included A.770041 (a Src family kinase inhibitor) and BX.795 (a PDK1 inhibitor). Previous studies indicated that Src family kinases are involved in cell adhesion, migration, and epithelial–mesenchymal transition, playing a critical role in cervical carcinogenesis (75). PDK1, a key kinase in the PI3K/AKT signaling pathway, is associated with tumor proliferation and drug resistance when aberrantly expressed (76), and its inhibition synergistically enhances the efficacy of chemotherapy or radiotherapy (77). These mechanisms provide a theoretical basis for the potential application of these drugs in cervical cancer treatment. Although these drugs are not yet approved for clinical use, our findings suggest that high-risk patients might benefit from these agents, warranting further investigation. Among other drugs demonstrating efficacy in the LRG, BIBW2992 (afatinib), an EGFR tyrosine kinase inhibitor, exhibited antitumor activity in tumors with activated EGFR pathways in prospective studies (78), further supporting the observed sensitivity of patients in the LRG to EGFR inhibitors. Overall, the drug sensitivity results in this study suggest a possible association between risk stratification and predicted responses to several candidate drugs and provide exploratory leads for subsequent drug screening and mechanistic studies. However, these results were based on computational inference from GDSC cell line drug response data and the pRRophetic algorithm, and they cannot be used to determine the actual efficacy or clinical benefit of specific drugs in patients with CSCC across different risk groups. Therefore, these candidate drugs and their potential risk group specificity require further validation through cellular and animal experiments, independent drug response cohorts, and prospective clinical studies.

This study had several limitations. The data were derived from public databases, and the data were retrospectively analyzed, which might have led to sample selection bias and missing information. In addition, the proposed model was only validated in a single dataset, and its AUC was only moderate. Therefore, the generalizability of the conclusions must be further verified through multicenter prospective cohort studies. Second, although the differential expression of the four key genes was verified in cell lines, functional experiments, such as gene knockout/overexpression and *in vivo* tumorigenesis assays, were lacking, and thus, their direct driving roles in the progression of CSCC remain unclear. Follow-up studies are required to deeply explore the underlying mechanism through cell and animal models. Furthermore, the MAM-related mechanistic component of this study was essentially hypothesis-generating. The identified prognostic genes are not known core regulators of MAM, and their associations with MAM were inferred primarily from the existing literature. The current experimental validation supports only the differential expression of the four candidate genes in CSCC cells, and they cannot directly establish a causal or mechanistic relationship between changes in manganese levels and gene expression. Further studies are needed to verify this association through ICP-MS detection of manganese ion concentrations and chelation/supplementation experiments, and more core metabolic genes should be included to optimize the proposed model. Finally, because of the limited clinical information in the TCGA and GEO databases, key variables including FIGO stage and treatment regimen were not included in the analysis, which might have limited the accurate evaluation of the independent prognostic value of the risk model. These issues need to be further addressed in prospective cohorts with more complete clinical data.

## 5 Conclusions

In this study, we identified four prognostically relevant key genes associated with a previously defined MRG set (*PARP1*, *SLC20A1*, *GALNTL6*, and *TAGLN*) through bioinformatic analysis and subsequently constructed a predictive risk model. The model demonstrated good performance in patient risk stratification, immune infiltration characteristics, and drug sensitivity, underscoring its potential clinical utility. Our findings provide new insights into the role of MAM in CSCC. However, the conclusions were based primarily on bioinformatic analyses, and these four genes are not currently established core regulators of MAM. Therefore, further validation in larger clinical cohorts is required. Future studies should focus on elucidating the molecular mechanisms by which these four genes regulate MAM and tumor progression, thereby providing a more robust theoretical basis for the precision diagnosis and treatment of CSCC.

## Data Availability

The original contributions presented in the study are included in the article/**Supplementary Material**. Further inquiries can be directed to the corresponding authors.

## Glossary

CSCC: Cervical squamous cell carcinoma
MAM: Manganese metabolism
MRGs: Manganese metabolism-related genes
DEGs: Differentially expressed genes
WGCNA: Weighted gene co-expression network analysis
GO: Gene Ontology
BP: Biological Process
CC: Cellular Component
MF: Molecular Function
KEGG: Kyoto Encyclopedia of Genes and Genomes
PPI: Protein-protein interaction
DFS: Disease-free survival
HR: Hazard Ratio
LASSO: Least absolute shrinkage and selection operator
TCGA-CSCC: The Cancer Genome Atlas - cervical squamous cell carcinoma
ROC: Receiver operating characteristic
AUC: Area under the curve
HRG: High-risk group
LRG: Low-risk group
KM: Kaplan-Meier
GSEA: Gene set enrichment analysis
ssGSEA: Single-sample gene set enrichment analysis
IC_50_: Half-maximal inhibitory concentration
GDSC: Genomics of Drug Sensitivity in Cancer
TFs: Transcription factors
cDNA: Complementary DNA
qRT-PCR: Quantitative reverse transcription polymerase chain reaction
GAPDH: Glyceraldehyde-3-phosphate dehydrogenase
WB: Western blot
IF: Immunofluorescence
PBS: Phosphate-buffered saline
PFA: Paraformaldehyde
BSA: Bovine serum albumin
DAPI: 4’,6-diamidino-2-phenylindole
SD: Standard deviation
ANOVA: Analysis of variance
EMT: Epithelial-mesenchymal transition
TKI: Tyrosine kinase inhibitor
VEGFR: Vascular endothelial growth factor receptor
PDGFR: Platelet-derived growth factor receptor
EGFR: Epidermal growth factor receptor.
